# Discovery of solabiose phosphorylase and its application for enzymatic synthesis of solabiose from sucrose and lactose

**DOI:** 10.1038/s41598-021-04421-2

**Published:** 2022-01-07

**Authors:** Wataru Saburi, Takanori Nihira, Hiroyuki Nakai, Motomitsu Kitaoka, Haruhide Mori

**Affiliations:** 1 Research Faculty of Agriculture, Hokkaido University, Kita 9, Nishi 9, Sapporo, 060-8589 Japan; 2 Faculty of Agriculture, Niigata University, Niigata, 950-2181 Japan

**Keywords:** Biochemistry, Biotechnology, Microbiology

## Abstract

Glycoside phosphorylases (GPs), which catalyze the reversible phosphorolysis of glycosides, are promising enzymes for the efficient production of glycosides. Various GPs with new catalytic activities are discovered from uncharacterized proteins phylogenetically distant from known enzymes in the past decade. In this study, we characterized *Paenibacillus borealis* PBOR_28850 protein, belonging to glycoside hydrolase family 94. Screening of acceptor substrates for reverse phosphorolysis, in which α-d-glucose 1-phosphate was used as the donor substrate, revealed that the recombinant PBOR_28850 produced in *Escherichia coli* specifically utilized d-galactose as an acceptor and produced solabiose (β-d-Glc*p*-(1 → 3)-d-Gal). This indicates that PBOR_28850 is a new GP, solabiose phosphorylase. PBOR_28850 catalyzed the phosphorolysis and synthesis of solabiose through a sequential bi-bi mechanism involving the formation of a ternary complex. The production of solabiose from lactose and sucrose has been established. Lactose was hydrolyzed to d-galactose and d-glucose by β-galactosidase. Phosphorolysis of sucrose and synthesis of solabiose were then coupled by adding sucrose, sucrose phosphorylase, and PBOR_28850 to the reaction mixture. Using 210 mmol lactose and 280 mmol sucrose, 207 mmol of solabiose was produced. Yeast treatment degraded the remaining monosaccharides and sucrose without reducing solabiose. Solabiose with a purity of 93.7% was obtained without any chromatographic procedures.

## Introduction

Glycoside phosphorylases (GPs) catalyze the reversible phosphorolysis of glycosides with net retention or inversion of the anomeric configuration of substrates^1^. In the classification of carbohydrate active enzymes based on amino acid sequence similarity, known GPs are classified into the glycoside hydrolase (GH) and glycosyltransferase families^2,3^. GPs selectively and efficiently produce oligosaccharides from sugar 1-phosphate and the acceptor sugar^1^. Coupling two types of GPs provides a synthetic system for the production of a target sugar from abundant sugars. For example, in the coupling maltose phosphorylase (EC 2.4.1.8) and trehalose phosphorylase (EC 2.4.1.64), maltose phosphorylase phosphorolyzes maltose to β-d-glucose 1-phosphate and d-glucose, leading to the production of trehalose from these two compounds by trehalose phosphorylase^4^. Furthermore, by combining other types of enzymes, such as mutase and epimerase, synthesis of d-galactoside and d-mannoside from sucrose are achieved^5–7^. In order to expand the application of GPs for sugar synthesis, new GPs need to be discovered.

GH family 94 (GH94) mainly comprises β-d-glucoside-acting inverting GPs, including cellobiose phosphorylase (EC 2.4.1.20; CBP), laminaribiose phosphorylase (EC. 2.4.1.31; LBP), cellodextrin phosphorylase (EC 2.4.1.49; CDP), *N*,*N’*-diacetylchitobiose phosphorylase (EC 2.4.1.280; ChBP), cellobionic acid phosphorylase (EC 2.4.1.321; CBAP), 1,2-β-oligoglucan phosphorylase (EC 2.4.1.333; 12BOGP), and product-length regulatory C-terminal domain (12BOGP-like) of dual-function cyclic β-1,2-glucan synthase (EC 2.4.1.-; CB12GS)^8^. Members of this family share an (α/α)_6_-barrel catalytic domain and an N-terminal β-sandwich domain^9,10^. The conserved Asp residue on the loop connecting the 5th and 6th α-helices of the catalytic domain (α5 → α6 loop) serves as the general acid catalyst for the phosphorolysis of the substrate. The catalytic Asp donates a proton to the glycosidic oxygen of the substrate, and the inorganic phosphate attacks anomeric carbon to produce sugar phosphate in an inverted anomeric configuration.

**Figure 1:**
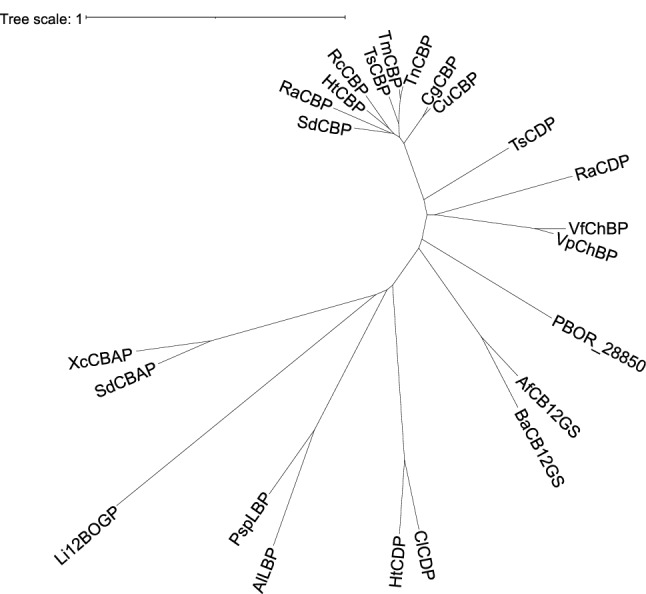
Phylogenetic tree of GH94 enzymes. Multiple sequence alignment was carried out by MAFFT^31^, and phylogenetic tree was constructed using Interactive Tree of Life version 5.7 (https://itol.embl.de). SdCBP, *Saccharophagus degradans* CBP (ABD80580.1); RaCBP, *Ruminococcus albus* CBP (ADU20744.1); HtCBP, *Hungateiclostridium thermocellum* CBP (ABN51514.1); RcCBP, *Ruminiclostridium cellulolyticum* CBP (ACL76454.1); TsCBP, *Thermoclostridium stercorarium* CBP (AAC45510.1); TmCBP, *Thermotoga maritima* CBP (AAD36910.1); TnCBP, *Thermotoga neapolitana* CBP (AAB95491.2); CgCBP, *Cellulomonas gilvus* CBP (BAA28631.1); CuCBP, *Cellulomonas uda* CBP (AAQ20920.1); TsCDP, *T. stercorarium* CDP (AAC45511.1); RaCDP, *R. albus* CDP (ADU22883.1); VfChBP, *Vibrio furnissii* ChBP (AAG23740.1); VpChBP, *Vibrio proteolyticus* ChBP (BAC87867.1); AfCB12GS, *Agrobacterium fabrum* CB12GS (AAK73356.1); BaCB12GS, *Brucella abortus* CB12GS (ACD71661.1); ClCDP, *Cellulosilyticum lentocellum* CDP (ADZ85667.1); HtCDP, *H. thermocellum* CDP (ABN54185.1); AlLBP, *Acholeplasma laidlawii* LBP (ABX81345.1); PspLBP, *Paenibacillus* sp. YM1 LBP (BAJ10826.1); Li12BOGP, *Listerila innocua* 12BOGP; SdCBAP, *S. degradans* CBAP (ABD80168.1); XcCBAP, *Xanthomonas campestris* CBAP (AAM43298.1). GenBank accession numbers are shown in parenthesis.

Rapidly increasing information on genome sequences has made it possible to screen novel enzymes based on the phylogenetic analysis of putative proteins. In GH94, CBAP and 12BOGP were discovered using this approach^11,12^. A putative GH94 protein PBOR_28850 (GenBank accession number, AIQ60507.1) from *Paenibacillus borealis* DSM 13188 is phylogenetically distant from any characterized enzymes of GH94, and its sequence identity with the characterized GH94 enzymes is 11.5–29.2% (Fig. 1). In this study, we characterized the enzymatic functions of PBOR_28850 and found that it can phosphorolyze solabiose (β-d-Glc*p*-(1 → 3)-d-Gal), which was originally identified in the sugar part of solanine from *Solanum tuberosum*^13^.

## Results

### Screening of substrates with PBOR_28850

**Figure 2:**
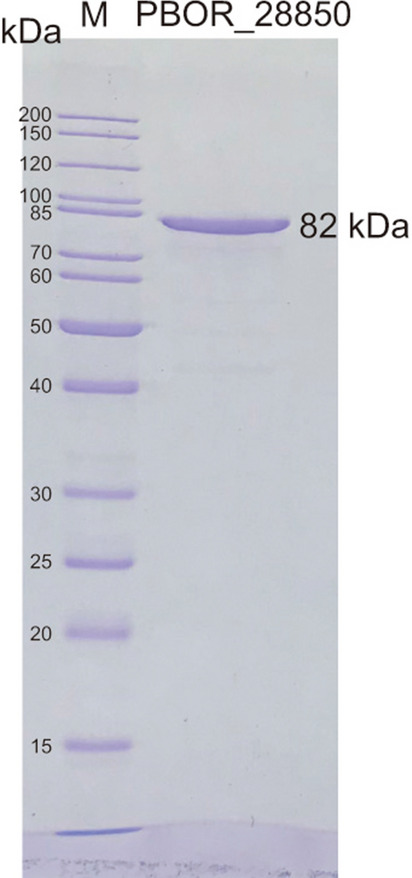
SDS-PAGE of purified PBOR_28850. One microgram of purified PBOR_28850 was analyzed. Lane M, standard of molecular mass. The image of the upper and lower parts of the gel was cropped. The gel image was cropped vertically to eliminate redundant part, but not horizontally. Unprocessed image is shown in Supplementary Fig. S1.

Recombinant PBOR_28850 with a C-terminal His-tag (6 His residues were attached to Met768) was produced in an *Escherichia coli* transformant and purified via Ni-affinity and gel filtration column chromatography. From *E. coli* cells harvested from 1 L of the culture broth, 38.9 mg of purified protein was obtained. The purified PBOR_28850 showed a single band of 82 kDa on an SDS–polyacrylamide gel (Fig. 2). This molecular mass matched well with the value estimated from the amino acid sequence (85.9 kDa).

In the synthetic reactions with α-d-glucose 1-phosphate (α-Glc1*P*) and various sugars, PBOR_28850 liberated inorganic phosphate only in the presence of d-galactose. The rate of the reaction with d-galactose and α-Glc1*P* was 3.23 ± 0.23 s^−1^ at 30 °C and pH 6.5, while that for the other acceptor sugars tested was < 0.1 s^−1^.

### Isolation and structural analysis of the reaction product using PBOR_28850

**Figure 3:**
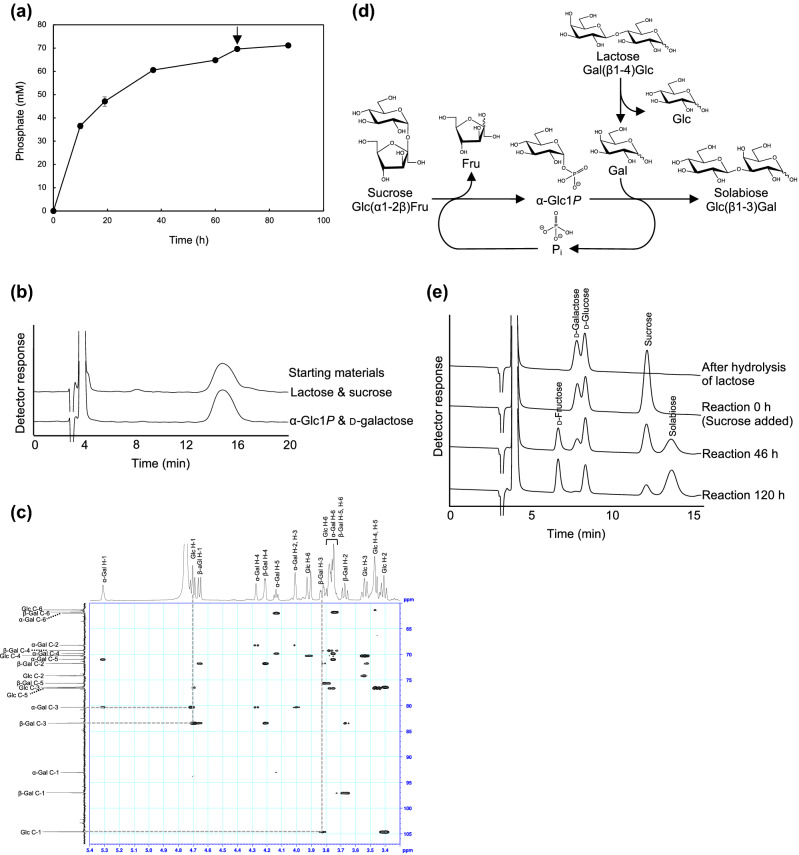
Enzymatic synthesis of solabiose. **(a)** Reaction time course of the reaction of PBOR_28850 with 100 mM α-Glc1*P* and d-galactose. Arrow indicates the time of adding the additional enzyme. Values and error bars indicate average and standard deviation for three independent experiments, respectively. **(b)** HPLC elution profile of solabiose prepared from α-Glc1*P* and d-galactose and from lactose and sucrose. **(c)** Heteronuclear multiple bond correlation spectroscopy analysis of solabiose produced from α-Glc1*P* and d-galactose. Dotted line indicates correlation signals between β-d-glucosyl residue and d-galactose residue. **(d)** Reaction scheme of enzymatic synthesis of solabiose from lactose and sucrose. β-Galactosidase hydrolyzes lactose to d-galactose and d-glucose. SP phosphorolyzes sucrose to α-Glc1*P* and d-fructose. α-Glc1*P* and d-galactose are used in the reverse phosphorolysis catalyzed by PBOR_28850 to produce solabiose. Inorganic phosphate used in the phosphorolysis of sucrose is generated in the reverse phosphorolysis of solabiose. **(e)** HPLC elution profile of the reaction samples.

The reaction of PBOR_28850 with α-Glc1*P* and d-galactose was monitored by measuring the amount of inorganic phosphate liberated at the same molar concentration as the sugar product (Fig. 3a). The inorganic phosphate concentration reached 65 mM after 60 h. Supplementation of the enzyme at this point slightly increased the inorganic phosphate concentration to 70 mM after further incubation for 8 h (68 h from the reaction start). The inorganic phosphate concentration remained stable during the reaction period from reaction 68 h to 87 h (71 mM at 87 h).

The reaction product from α-Glc1*P* and d-galactose was produced at an 11 mL scale (1.1 mmol of α-Glc1*P* and d-galactose). The concentration of the liberated inorganic phosphate was 60 mM after a 24 h reaction. From this reaction mixture, 200 mg of the product was purified via gel-filtration column chromatography. The purity of the product was 99.4%, which was estimated from the relative peak area in the HPLC analysis (Fig. 3b). The signal at 365.11 *m/z* [M + Na]^+^ was obtained by electrospray ionization mass spectrometry analysis, and the product was determined to be a disaccharide. In the heteronuclear multiple bond correlation spectroscopy analysis of the product, correlation signals between H-1 of the d-glucosyl residue and C-3 of the d-galactose residue, and between C-1 of the d-glucosyl residue and H-3 of the β-d-galactose residue were detected (Fig. 3c). This indicates that the glycosidic linkage was formed between C-1 of the d-glucosyl residue and C-3 of the d-galactose residue. As the *J* value of H-1 of the d-glucosyl residue was 7.55 Hz, the d-glucosyl residue was linked to d-galactose through a β-linkage. Thus, the reaction product was determined to be solabiose (β-d-Glc*p*-(1 → 3)-d-Gal). The molar ratio of the α- and β-anomers of solabiose, estimated from the signal intensity of H-1 of the reducing-end d-galactose residue in ^1^H-NMR, was 1:3. This molar ratio is consistent with that of d-galactose^14^. The chemical shifts of solabiose are as follows: ^1^H-NMR (D_2_O) *δ* 5.31 (s, α-Gal H-1), 4.70 (d, *J* = 7.55 Hz, Glc H-1), 4.66 (d, *J* = 7.90 Hz, β-Gal H-1), 4.27 (s, α-Gal H-4), 4.21 (d, *J* = 2.85 Hz, β-Gal H-4), 4.14 (t, *J* = 6.15 Hz, α-Gal H-5), 4.01 (m, α-Gal H-2), 4.01 (m, α-Gal H-3), 3.92 (d, *J* = 12.4 Hz, Glc H-6), 3.84 (m, β-Gal H-3), 3.79 (m, β-Gal H-6), 3.78 (m, Glc H-6), 3.78 (m, α-Gal H-6), 3.75 (m, β-Gal H-5), 3.67 (t, *J* = 8.5 Hz, β-Gal H-2), 3.55 (m, Glc H-3), 3.48 (m, Glc H-5), 3.46 (m, Glc H-4), 3.41 (t, *J* = 8.4 Hz, Glc H-2); ^13^C-NMR (D_2_O) *δ* 104.6 (Glc C-1), 97.0 (β-Gal C-1), 93.0 (α-Gal C-1), 83.4 (β-Gal C-3), 80.3 (α-Gal C-3), 76.5 (Glc C-5), 76.4 (Glc C-3), 75.6 (β-Gal C-5), 74.2 (Glc C-2), 71.8 (β-Gal C-2), 71.0 (α-Gal C-5), 70.3 (Glc C-4), 69.8 (α-Gal C-4), 69.2 (β-Gal C-4), 68.2 (α-Gal C-2), 62.0 (α-Gal C-6), 61.8 (β-Gal C-6), 61.4 (Glc C-6).

### Enzymatic characteristics of PBOR_28850

**Figure 4:**
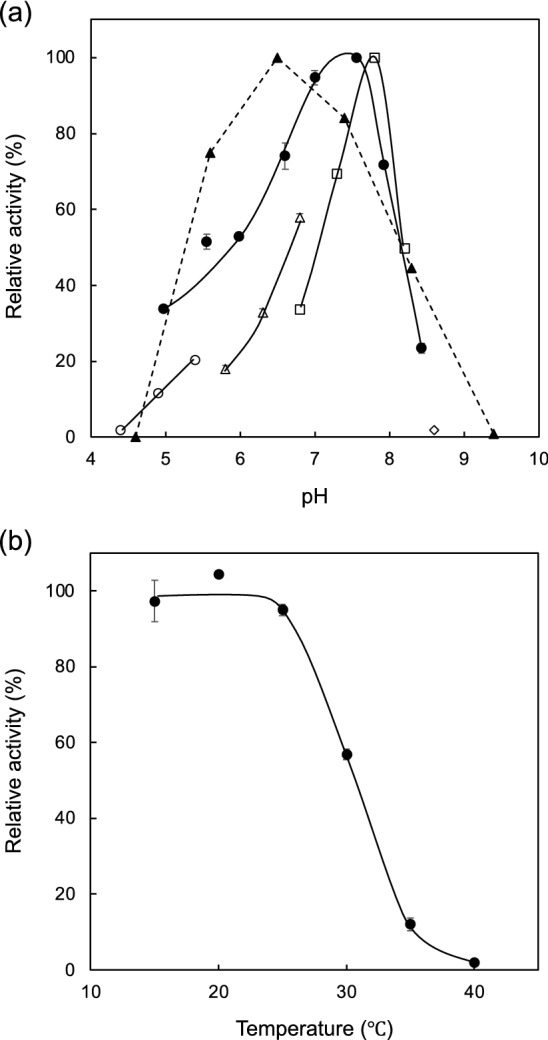
Effects of pH and temperature. **(a)** Effect of pH. Closed circles show activity of the phosphorolysis. Open symbols indicate activity of reverse phosphorolysis. Circles, triangles, squares, and diamond show the reaction in sodium acetate buffer, MES-NaOH buffer, HEPES–NaOH buffer, and glycine–NaOH buffer, respectively. Closed triangles show residual activity after the pH treatment at pH 4.6–9.4 at 4 °C for 24 h. **(b)** Temperature stability. Residual activity after the heat treatment at 15–40 °C for 15 min is shown. Values and error bars indicate average and standard deviation for three independent experiments, respectively.

**Figure 5:**
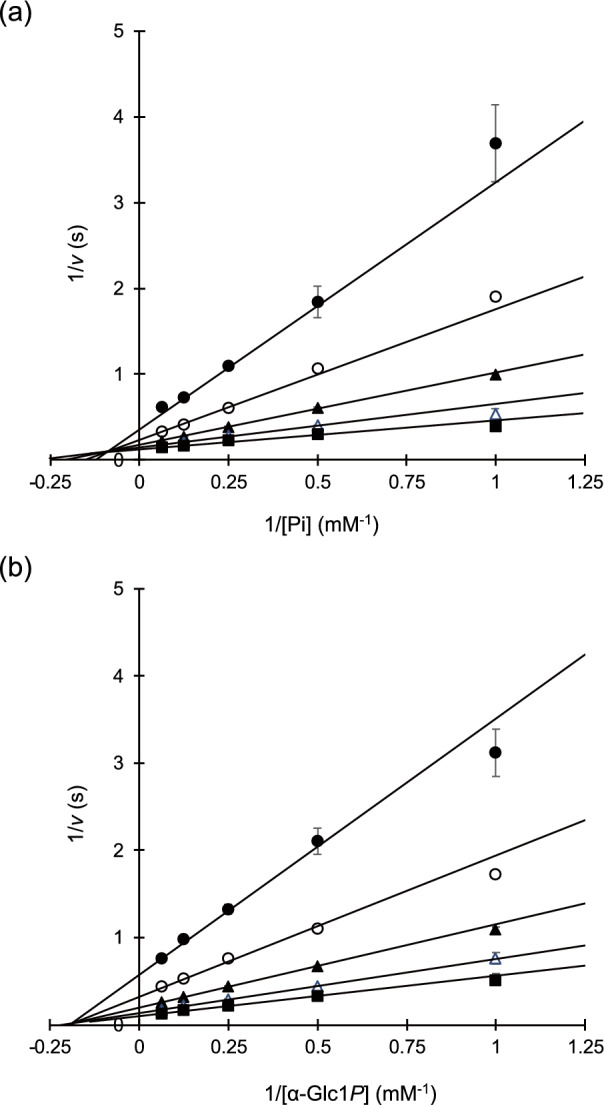
Double reciprocal plots for the phosphorolysis and synthesis of solabiose. **(a)** Phosphoroylsis of solabiose. Reaction rates at 1, 2, 4, 8, and 16 mM inorganic phosphate in the presence of 1 (filled circles), 2 (open circles), 4 (filled triangles), 8 (open triangles), and 16 mM solabiose (filled squares) are shown. **(b)** Synthesis of solabiose. Reaction rates at 1, 2, 4, 8, and 16 mM α-Glc1*P* in the presence of 1 mM (filled circles), 2 mM (open circles), 4 mM (filled triangles), 8 mM (open triangles), and 16 mM d-galactose (filled squares) are shown. Average of three independent experimental values are plotted. Error bars indicate standard deviation of the values.

The optimum pH of PBOR_28850 for the phosphorolysis and synthesis of solabiose was determined based on the reaction velocities at various pH values. PBOR_28850 showed the highest activity at pH 7.6 and pH 7.8 in the phosphorolysis and synthesis of solabiose, respectively (Fig. 4). This enzyme retained more than 85% of its original activity in a pH range of 6.5–7.4 (at 4 °C for 24 h) and below 25 °C (at pH 8.0 for 15 min). As PBOR_28850 produced inorganic phosphate linearly during the 10 min reaction at 30 °C with 1 mM α-Glc1*P* and d-galactose (the lowest substrate concentration used in this study), kinetic analysis was conducted at 30 °C. The reaction velocity for the phosphorolysis and synthesis of solabiose was measured at various substrate concentrations (Fig. 5). The reaction rates for both directions of the reaction obeyed the rate equation of the sequential bi-bi mechanism. Kinetic parameters for the phosphorolysis of solabiose were calculated to be: *k*_cat_, 9.14 ± 0.11 s^-1^; *K*_mA_, 1.53 ± 0.05 mM; *K*_mB_, 2.26 ± 0.27 mM; and *K*_iA_, 11.1 ± 1.7 mM (A and B represent inorganic phosphate and solabiose, respectively). Kinetic parameters for the synthesis of solabiose were: *k*_cat_, 14.5 ± 0.3 s^-1^; *K*_mA_, 4.27 ± 0.16 mM; *K*_mB_, 7.35 ± 0.23 mM; and *K*_iA_, 5.21 ± 0.18 mM (A and B represent α-Glc1*P* and d-galactose, respectively).

### Synthesis of solabiose from lactose and sucrose

Enzymatic synthesis of solabiose from the abundant sugars lactose and sucrose was established. To produce d-galactose, 300 mM lactose (210 mmol) was hydrolyzed by β-galactosidase (EC 3.2.1.23). After denaturation of the β-galactosidase by heating, sucrose (280 mmol), sucrose phosphorylase (SP; EC 2.4.1.7), and PBOR_28850 were added (concentrations of d-galactose and sucrose were 263 and 350 mM, respectively), and the phosphorolysis of sucrose by SP and the synthesis of solabiose by PBOR_28850 were coupled (Fig. 3d). After a reaction time for 120 h, almost all the d-galactose was consumed, and the concentration of solabiose reached 259 mM (207 mmol), indicating that the yield of solabiose was 98.6% (Fig. 3e). Yeast treatment was also performed, which showed that yeast consumed d-fructose, d-glucose, and sucrose in the reaction mixture without decreasing the solabiose content. The purity of solabiose in the reaction mixture increased from 44.2% (after the reaction for 120 h) to 93.7% (Fig. 3b).

### Prediction of substrate binding mechanism of PBOR_28850

**Figure 6:**
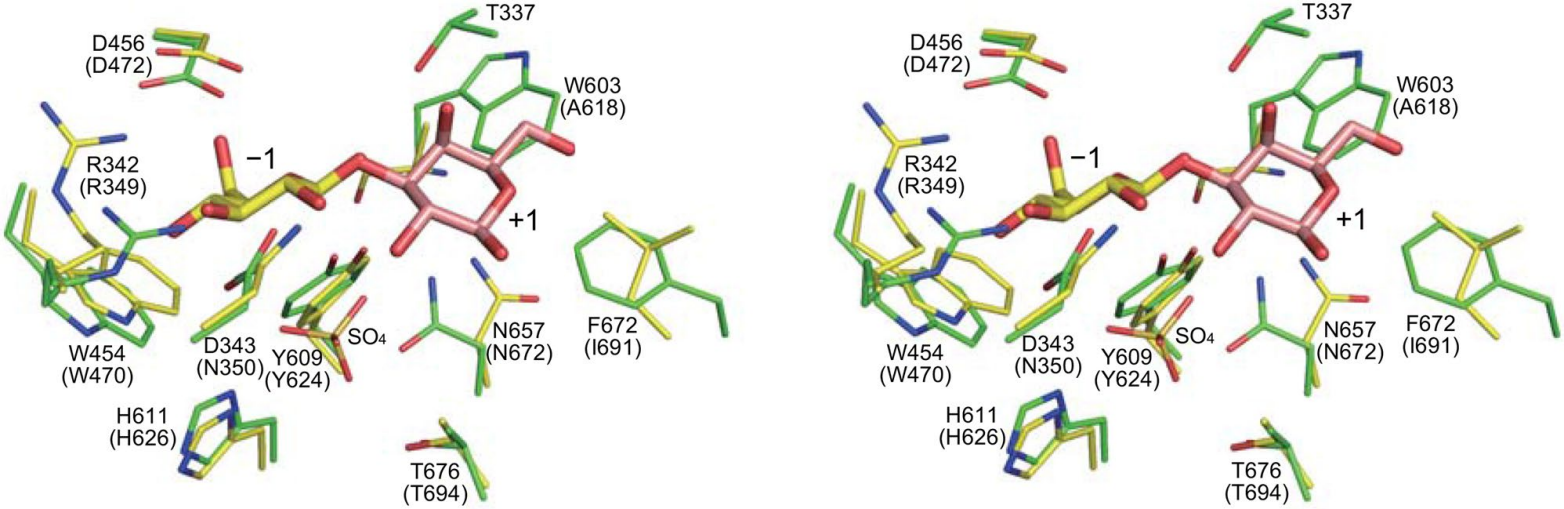
Predicted structure of PBOR_28850. Structure of PBOR_28850 was predicted by AlphaFold^26^. The model structure of PBOR_28850 (green) is superimposed onto *S. degradans* CBAP in complex with 3-*O*-β-d-glucopyranosyl-α-d-glucopyranuronic acid (yellow; PDB entry, 4ZLI). To predict the binding mode of solabiose in PBOR_28850, β-d-galactose in ideal form (PDB entry, GAL), shown in pale orange, is overlapped onto the α-d-glucopyranuronic acid residue (α-d-glucopyranuronic acid residue is not shown).

**Figure 7:**
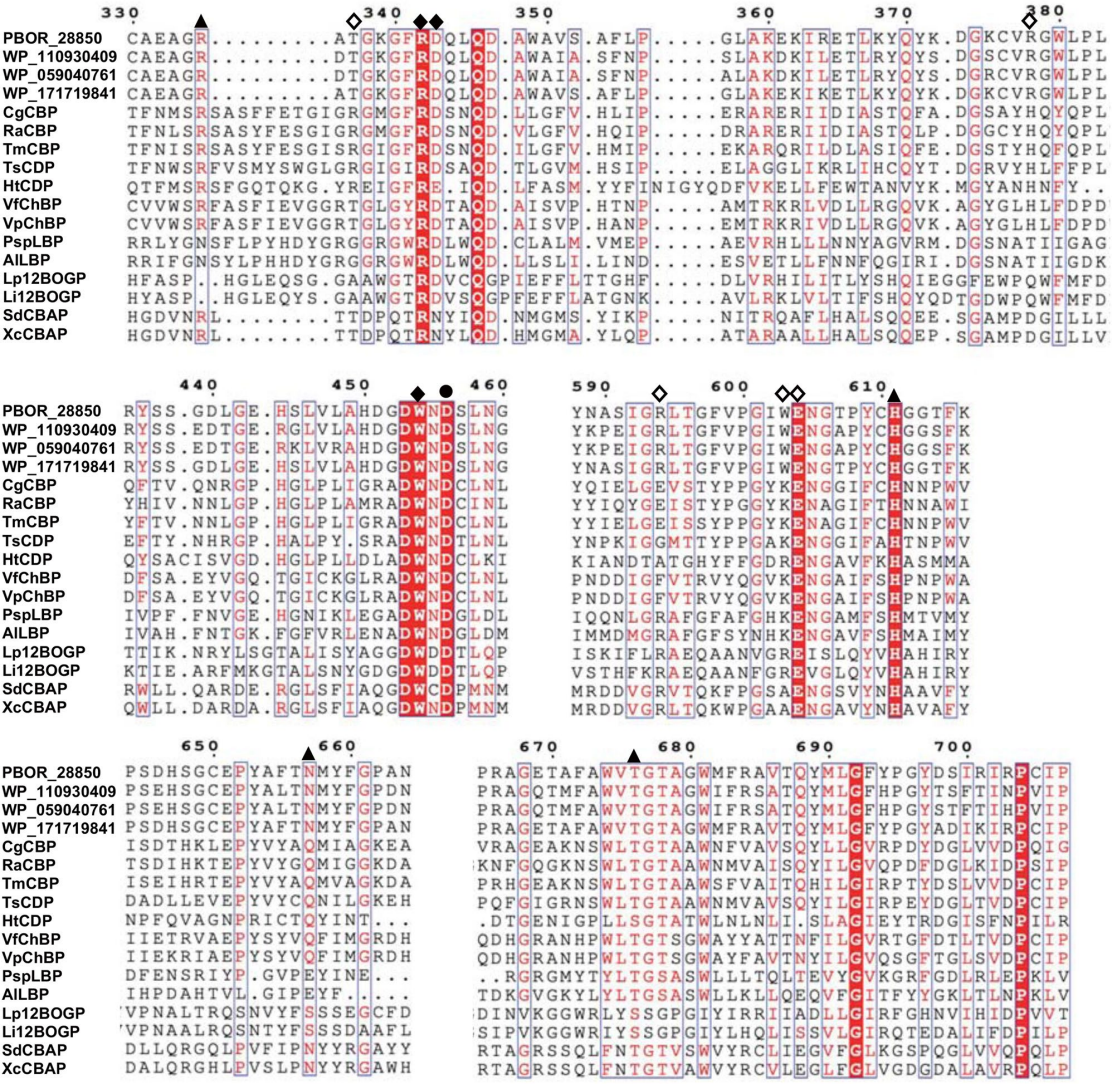
Comparison of amino acid sequences of GH94 enzymes. Multiple sequence alignment was constructed using MAFFT-DASH^31^. Filled circle indicates the general acid catalyst for the phosphorolysis of substrates. The residues shown by filled triangles are responsible for binding to phosphate. Filled and open diamonds indicate residues involved in the formation of subsites −1 and + 1, respectively. Amino acid numbers of PBOR_28850 are shown above the figure. WP_110930409, WP_059040761, and WP_WP_171719841 from *P. bouchesdurhonensis*, *P. rubinfantis*, and *P. phytohabitans*, respectively, are homologues of PBOR_28850. CgCBP, *C. gilvus* CBP; RaCBP, *R. albus* CBP; TmCBP, *T. maritima* CBP; TsCDP, *T. stercorarium* CDP; HtCDP, *H. thermocellum* CDP; VfChBP, *V. furnissii* ChBP; VpChBP, *V. proteolyticus* ChBP; PspLBP, *Paenibacillus* sp. YM1 LBP; AlLBP, *A. laidlawii* LBP; Lp12BOGP, *Lachnoclostridium phytofermentans* 12BOGP (GenBank number, ABX41081.1); Li12BOGP, *L. innocua* 12BOGP; SdCBAP, *S. degradans* CBAP; and XcCBAP, *X. campestris* CBAP.

To predict the substrate-binding mechanism, a model structure of PBOR_28850 was constructed using AlphaFold^15^. This model structure was superimposed onto *Saccharophagus degradans* CBAP in complex with 3-*O*-β-d-glucopyranosyl-α-d-glucopyranuronic acid (PDB entry, 4ZLI)^16^ (Fig. 6). The residues of PBOR_28850, responsible for the catalysis (Asp456) and substrate binding in subsite − 1 (Arg342, Asp343, Trp454, Tyr609, His611, and Thr676) are predicted to be spatially similar to the corresponding residues of CBAP. These residues are well conserved in GH94 enzymes (Asp343 is substituted by Asn350 in the CBAP, but most GH94 enzymes have Asp at this position) (Fig. 7). Consistent with the diversity of substrate specificity of GH94 enzymes, amino acid residues forming subsite + 1 are less conserved. To predict the binding mode of d-galactose in subsite + 1 of PBOR_28850, β-d-galactose in ideal form (PDB entory, GAL) was superimposed onto the α-d-glucopyranuronic acid residue bound to subsite + 1. Trp603 and Phe672 are predicted to be suitably located to interact with C-6 of the d-galactose residue of solabiose in subsite + 1. The axial hydroxy group at the C-4 position of d-galactose points to Thr337. These residues are conserved in the homologous proteins of PBOR_28850 from *Paenibacillus* species (Fig. 7).

## Discussion

GPs are promising biocatalysts for the efficient production of oligosaccharides. However, the number of known GPs is small; therefore, the discovery of new GPs is desirable. Owing to the rapid increase in genome information, GPs with new catalytic activity have recently been discovered from uncharacterized proteins that are phylogenetically distant from known enzymes^7,11,12,17–24^. In this study, we conducted biochemical characterization of an uncharacterized GH94 protein, PBOR_28850, from *P. borealis*, and identified this protein as a new GP, solabiose phosphorylase.

Solabiose was first isolated from the partial acid hydrolysate of *Solanum tuberosum*^13^. This disaccharide was also prepared from the extracellular polysaccharide of phytopathogenic *Xanthomonas* spp.^25^ and lipid-bound sugars from *Rhizobium meliloti*^26^. The solabiose structure is a part of the repeating unit of water-absorbing polysaccharide from *Oxalobacteraceae* bacterium: 4)Glc(β1–4)Man(β1–4)Glc(β1–4)[Gal(β1–3)Glc(β1–3)Gal(β1–3)]Glc(β1-^27^. Considering that these organisms and *P. borealis* are soil inhabitants, PBOR_28850 is presumably involved in the metabolism of solabiose, released from solabiose-containing compounds. The *pbor_28850* gene comprises a gene cluster together with *pbor_28840* and *pbor_28845*, encoding ATP-binding cassette transporter components, *pbor_28855*, encoding GH130 β-1,4-mannooligosaccharide phosphorylase (EC 2.4.1.319), *pbor_28860*, encoding multi-domain proteins including the GH26 endoglucanase-like catalytic domain and several carbohydrate-binding modules, and *pbor_28865*, encoding solute binding proteins. These proteins might contribute to the degradation of soil microbe exopolysaccharides containing β-(1 → 4)-d-mannosyl and solabiose parts, such as the *Oxalobacteraceae* exopolysaccharide^27^. Genes encoding homologous proteins of PBOR_28850 with 64.2–96.4% amino acid sequence identity, are distributed in several *Paenibacillus* species such as *Paenibacillus bouchesdurhonensis*, *Paenibacillus rubinfantis*, and *Paenibacillus phytohabitans*. This implies that these *Paenibacillus* strains could also utilize solabiose-containing compounds as a carbon source.

Substrate binding mechanism of PBOR_28850 was predicted based on the structural comparison of the model structure of PBOR_28850 and the crystal structure of the complex of *S. degradans* CBAP and 3-*O*-β-d-glucopyranosyl-α-d-glucopyranuronic acid. The residues of PBOR_28850, involved in the catalysis, phosphate binding, and sugar binding in subsite − 1, are conserved well as in GH94 enzymes, and predicted to be arranged similarly to those of the compared enzyme. This indicates that PBOR_28850 obeys the common catalytic mechanism of GH94 enzymes. In the prediction of d-galactose binding in subsite + 1 of PBOR_28850, Thr337 and two aromatic residues Trp603 and Phe672 are found as candidate binding residues. Considering that PBOR_28850 is very specific to d-galactose as acceptor substrate of the reverse phosphorolysis (this enzyme does not use d-glucose as acceptor), PBOR_28850 strictly recognizes axial 4-OH of d-galactose in subsite + 1 or equatorial 4-OH of d-glucose causes steric hindrance upon binding to subsite + 1. Since no amino acid residue, which causes steric hindrance against 4-OH of d-glucose, is not found in the model structure of PBOR_28850, Thr337, situated close to 4-OH of d-galactose, presumably has an essential interaction with d-galactose in subsite + 1.

In this study, we established an efficient enzymatic synthesis of solabiose from sucrose and lactose using PBOR_28850. As PBOR_28850 had very high acceptor specificity to d-galactose, solabiose was specifically produced from α-Glc1*P* and d-galactose, even in the presence of d-glucose and d-fructose as byproducts. As solabiose was not utilized as a carbon source for yeast, high purity solabiose was successfully obtained without any column chromatographic procedures. The yield of solabiose by this enzymatic synthesis is much higher than that of organic synthesis, as established previously^28^. Benzyl 2,6-di-*O*-acetyl-3-*O*-(2,3,4,6-tetra-*O*-acetyl-β-d-glucopyranosyl)-β-d-galactopyranoside was synthesized at a yield of 34% from benzyl 2,6-di-*O*-acetyl-β-d-galactopyranoside and tetra-*O*-acetyl-α-d-glucopyranosyl bromide, and solabiose was obtained from this compound through the removal of acetyl and benzyl groups. Furthermore, enzymatic synthesis is much simpler than organic synthesis and does not require any harmful reagents. The enzymatic synthesis of solabiose can easily be scaled up, making it possible to provide solabiose for physiological analysis in animals, plants, and microorganisms. The beneficial physiological functions of solabiose can be identified through such biological analyses.

## Methods

### Materials

d-Allose, d-glucose, α-Glc1*P*, d-gluconic acid, lactose, d-mannose, and d-xylose were purchased from Fujifilm Wako Pure Chemical (Osaka, Japan); *N*-acetyl-d-glucosamine, d-galactose, and sucrose were purchased from Nacalai Tesque (Kyoto, Japan); d-glucosamine was purchased from Tokyo Chemical Industry (Tokyo, Japan); cellobiose, d-galacturonic acid, and d-glucuronic acid were purchased from Sigma (St. Louis, MO, USA). β-(1 → 4)-Mannobiose was prepared as previously described^29^. Lactoless L3 (β-galactosidase) was provided by Daiwa Kasei (Shiga, Japan). SP from *Bifidobacterium longum* was prepared according to the method described by Nishimoto and Kitaoka^5^. One unit of SP was defined as the amount of enzyme required to phosphorolyze 1 μmol of sucrose in 1 min. SP activity was measured as follows: a reaction mixture (50 μL) containing appropriate concentration of enzyme, 20 mM sucrose, 40 mM sodium phosphate, 100 mM 4-(2-hydroxyethyl)-1-piperazineethanesulfonic acid (HEPES)-NaOH buffer (pH 7.0), and 0.2 mg/mL bovine serum albumin (BSA, Nacalai Tesque) was incubated at 37 °C for 10 min. The enzymatic reaction was terminated by incubating the sample at 80 °C for 3 min, and the liberated d-fructose was measured using a d-Fructose/d-Glucose Assay Kit (Megazyme, Brey, Ireland).

### Preparation of recombinant PBOR_28850

*pbor_28850* was amplified from the genomic DNA of *P. borealis* DSM 13188 (Deutsche Sammlung von Mikroorganismen und Zellkulturen, Braunschweig, Germany) using the primers (5′-ATGGCTGGCTTCGGCAATTTC-3′ and 5′-CTACATATCCACCTCTACAGTAT-3′) and KOD FX DNA polymerase (Toyobo, Osaka, Japan). The amplified DNA fragment was used as a template for subsequent PCR using the primers 5′-AAGAAGGAGATATACATATGGCTGGCTTCGGCAATTTC-3′ and 5′-CAGTGGTGGTGGTGGTGGTGCATATCCACCTCTACAGTAT-3′. The resulting DNA fragment was inserted into pET-23a (Novagene, Darmstadt, Germany) using the In-Fusion HD Cloning Kit (Takara Bio, Kusatsu, Japan). The inserted DNA and flanking regions were sequenced using Applied Biosystems 3130 Genetic Analyzer (Life Technologies, Carlsbad, CA, USA).

Transformants of *E. coli* BL21 (DE3) harboring the expression plasmid were cultured in 1 L of LB medium, containing 100 μg/mL ampicillin, at 37 °C until *A*_600_ reached 0.5. Production of the recombinant protein was induced by adding 0.1 mM isopropyl β-thiogalactoside, followed by incubation with vigorous shaking at 18 °C for 24 h. Bacterial cells, harvested via centrifugation (9600 × *g*, 4 °C, 10 min), were disrupted by sonication, and cell-free extracts were obtained by centrifugation (9600 × *g*, 4 °C, 10 min). Recombinant PBOR_28850 was purified from the extract via Ni affinity column chromatography using Chelating Sepharose Fast Flow (GE Healthcare, Uppsala, Sweden; 2.6 cm i.d. × 2.0 cm) equilibrated with 20 mM imidazole–HCl buffer (pH 7.0) containing 0.5 M NaCl. The adsorbed protein was eluted using a linear gradient of 20–500 mM imidazole (total elution volume, 200 mL) after elution of the non-adsorbed protein with the equilibration buffer. The collected sample was concentrated to 7.7 mg/mL by ultrafiltration using a Vivaspin YM-30 concentrator (Sartorius, Göttingen, Germany) and separated via gel filtration column chromatography using a Toyopearl HW-55S column (Tosoh, Tokyo, Japan; 2.6 cm i.d. × 100 cm) equilibrated with 10 mM HEPES–NaOH buffer (pH 7.0). Concentration of the purified enzyme was determined via amino acid analysis.

### Screening of acceptor substrate

The acceptor substrate of PBOR_28850 for reverse phosphorolysis was screened based on the rate of release of inorganic phosphate from α-Glc1*P* and various sugars. d-Allose, d-galactose, d-glucose, d-mannose, d-glucosamine, *N*-acetyl-d-glucosamine, d-gluconic acid, d-glucuronic acid, d-galacturonic acid, d-xylose, cellobiose, lactose, and β-(1 → 4)-mannobiose were tested as acceptors. A reaction mixture (50 μL) containing 72.4–3620 nM PBOR_28850, 20 mM α-Glc1*P*, 20 mM acceptor, and 0.2 M 2-(*N*-morpholino)ethanesulfonic acid (MES)-NaOH buffer (pH 6.5) was incubated at 30 °C for 10 min. The reaction was terminated by heating the sample at 80 °C for 3 min. The liberated Inorganic phosphate was measured as described by Lowry and Lopez^30^.

### Time course of reverse phosphorolysis with α-Glc1*P* and d-galactose

A reaction mixture containing 120 nM PBOR_28850, 0.1 M α-Glc1*P*, 0.1 M d-galactose, and 50 mM HEPES–NaOH buffer (pH 8.0) was incubated at 30 °C. After 60 h, 66 μL of 18.1 μM PBOR_28850 (the same amount of enzyme present at the start of the reaction) was added and the reaction mixture was further incubated at 30 °C. To monitor reaction progression, 100 μL of the reaction mixture was taken at the indicated time and incubated at 80 °C for 3 min to stop the reaction. Inorganic phosphate concentration was determined as described above.

### Preparation and structural analysis of oligosaccharide products

A reaction mixture (11 mL) containing 120 nM PBOR_28850, 0.1 M α-Glc1*P*, 0.1 M d-galactose, and 50 mM HEPES–NaOH buffer (pH 8.0) was incubated at 30 °C for 24 h. The oligosaccharide product was purified via gel filtration column chromatography using a Toyopearl HW-40S column (5.0 cm i.d. × 100 cm). Water was used as the mobile phase. The pooled fractions were desalted with Amberlite MB-4 (Organo, Tokyo, Japan) and lyophilized. The molecular masses of the products were measured via electrospray ionization mass spectrometry using an Exactive mass spectrometer (Thermo Scientific, San Jose, CA, USA). The sample was applied to a mass spectrometer by flow injection, using methanol as the mobile phase solvent. The positive ion was detected under following conditions: spray voltage, 3.00 kV; capillary temperature, 300 °C. NMR spectra were recorded in D_2_O (Sigma) at 27 °C using a Bruker AMX500 spectrometer (500 MHz; Bruker, Billerica, MA, USA). A series of two-dimensional homo- and heteronuclear correlated spectra (correlated spectroscopy, heteronuclear single quantum correlation spectroscopy, heteronuclear single quantum correlation total correlation spectroscopy, heteronuclear 2-bond correlation spectroscopy, and heteronuclear multiple bond correlation spectroscopy) were acquired to determine chemical structures of the reaction products.

### Standard enzyme assay

A reaction mixture (50 μL) containing an appropriate concentration of enzyme, 20 mM α-Glc1*P*, 20 mM d-galactose, and 0.2 M HEPES–NaOH buffer (pH 8.0) was incubated at 30 °C for 10 min. The reaction was terminated by incubating the sample at 80 °C for 3 min. The amount of liberated inorganic phosphate was measured as described above. One U of solabiose phosphorylase was defined as the amount of enzyme required to produce 1 μmol of solabiose in 1 min. The specific activity of purified PBOR_28850 was 8.90 U/mg.

### Optimum pH

The optimum pH of PBOR_28850 for the phosphorolysis and synthesis of solabiose was evaluated from the rate of the reaction at various pH values. The reaction rate for the phosphorolysis of solabiose was measured as follows: a reaction mixture (50 μL) containing 36.2 nM PBOR_28850, 20 mM solabiose, and 80 mM Britton-Robinson buffer (mixture of sodium acetate buffer, sodium phosphate buffer, and glycine–NaOH buffer; pH 5.0–8.4) was incubated at 30 °C for 10 min, and the generated d-galactose was measured using an l-Arabinose/d-Galactose Assay Kit (Megazyme) after stopping the reaction by heating at 80 °C for 3 min. Solabiose, synthesized from α-Glc1*P* and d-galactose, was used. The reaction rate for the synthesis of solabiose was measured as described above, except that sodium acetate buffer (pH 4.4–5.4), MES-NaOH buffer (pH 5.8–6.8), HEPES–NaOH buffer (pH 6.8–8.2), and glycine–NaOH buffer (pH 8.6) were used as reaction buffers.

### Stability range of pH and temperature

The stability range of pH and temperature was determined from the residual activity after the pH and temperature treatments, respectively. For the pH treatment, 9.05 μM PBOR_28850 was incubated in 250 mM buffer (pH 4.6–9.4) at 4 °C for 24 h. Sodium acetate buffer (pH 4.6–5.6), MES-NaOH buffer (pH 6.5), HEPES–NaOH buffer (pH 7.4–8.3), and glycine–NaOH buffer (pH 9.4) were used. For the temperature treatment, 201 nM PBOR_28850 was incubated in 336 mM HEPES–NaOH buffer (pH 8.0) containing 0.33 mg/mL BSA at 15–40 °C for 15 min. Residual activity was measured using the standard enzyme assay. The stability range was defined as the range in which the enzyme retained more than 85% of its original activity.

### Kinetic parameters for the phosphorolysis and synthesis of solabiose

For the phosphorolysis of solabiose, 50 μL of a reaction mixture containing 72.4 nM PBOR_28850, 1–16 mM solabiose, and 1–16 mM sodium phosphate, 0.2 M HEPES–NaOH buffer (pH 7.5), and 0.2 mg/mL BSA was incubated at 30 °C for 10 min, and the generated d-galactose was measured as described above. For the synthesis of solabiose, the rate of release of inorganic phosphate from 1 to 16 mM α-Glc1*P* and 1–16 mM d-galactose was measured using the standard enzyme assay. The reaction equation for the sequential bi-bi mechanism (Eq. 1) was fitted to the reaction rates at various substate concentrations using Grafit version 7.0.2 (Erithacus Software, East Grinstead, UK).

1 $$ v = k_{{{\text{cat}}}} \left[ {{\text{E}}_{0} } \right]\left[ {\text{A}} \right]\left[ {\text{B}} \right]/\left( {\left[ {\text{A}} \right]\left[ {\text{B}} \right] \, + K_{{{\text{mB}}}} \left[ {\text{A}} \right] \, + K_{{{\text{mA}}}} \left[ {\text{B}} \right] \, + K_{{{\text{iA}}}} K_{{{\text{mB}}}} } \right) $$

### Synthesis of solabiose from lactose and sucrose

A reaction mixture (700 mL) containing 300 mM lactose, 20 mM sodium phosphate buffer (pH 7.0), and 2% (v/v) Lactoless L3 was incubated at 30 °C for 21 h, and the reaction was stopped by heating the sample up to 85 °C using microwaves. After cooling the sample, 95.8 g of sucrose, 7 mL of 153 U/mL SP, and 20 mL of 44.8 U/mL PBOR_28850 were added to the mixture (increasing the volume of the reaction mixture to 800 mL) and the reaction mixture was incubated at 30 °C for 120 h. To consume the remaining sucrose and monosaccharides in the reaction mixture, 4.6 g of dry yeast (Nisshin Foods, Tokyo, Japan) was added, and the sample was incubated at 30 °C for 24 h with gentle shaking. The supernatant was obtained by centrifugation (9600 × *g*, 4 °C, 10 min) and decolored by incubation at 60 °C for 2 h in the presence of 3 g/L of active carbon (Nacalai Tesque). The sample was filtered with Celite No. 545 (Fujifilm Wako Pure Chemical) and deionized using Amberlite MB-4. The sample was filtrated through a 0.45 μm membrane filter (Advantec, Tokyo, Japan) and concentrated to 200 mL in vacuo.

### HPLC analysis of sugar content

The sugar content of the samples (10 mg/mL) was analyzed via HPLC under the following conditions: sample injection volume, 10 μL; column, Hilicpak VG-50 4E (Shodex, Tokyo, Japan; 4.6 mm i.d. × 250 mm); column temperature, 40 °C; eluent, acetonitrile/methanol/water = 75/20/5 (v/v/v); flow rate, 1 mL/min; detection, refractive index.

## Supplementary Information

Supplementary Figure S1.

## Data Availability

The datasets generated and/or analyzed in this study are available from the corresponding author upon reasonable request.
