# Supplementary material for: Discovery of solabiose phosphorylase and its application for enzymatic synthesis of solabiose from sucrose and lactose
Source: Sci Rep. 2022 Jan 7;12:259. doi: 10.1038/s41598-021-04421-2 (PMC8741936; doi:10.1038/s41598-021-04421-2)
Supplement: Supplementary file 1 — Supplementary Figure S1. [file 41598_2021_4421_MOESM1_ESM.pdf]

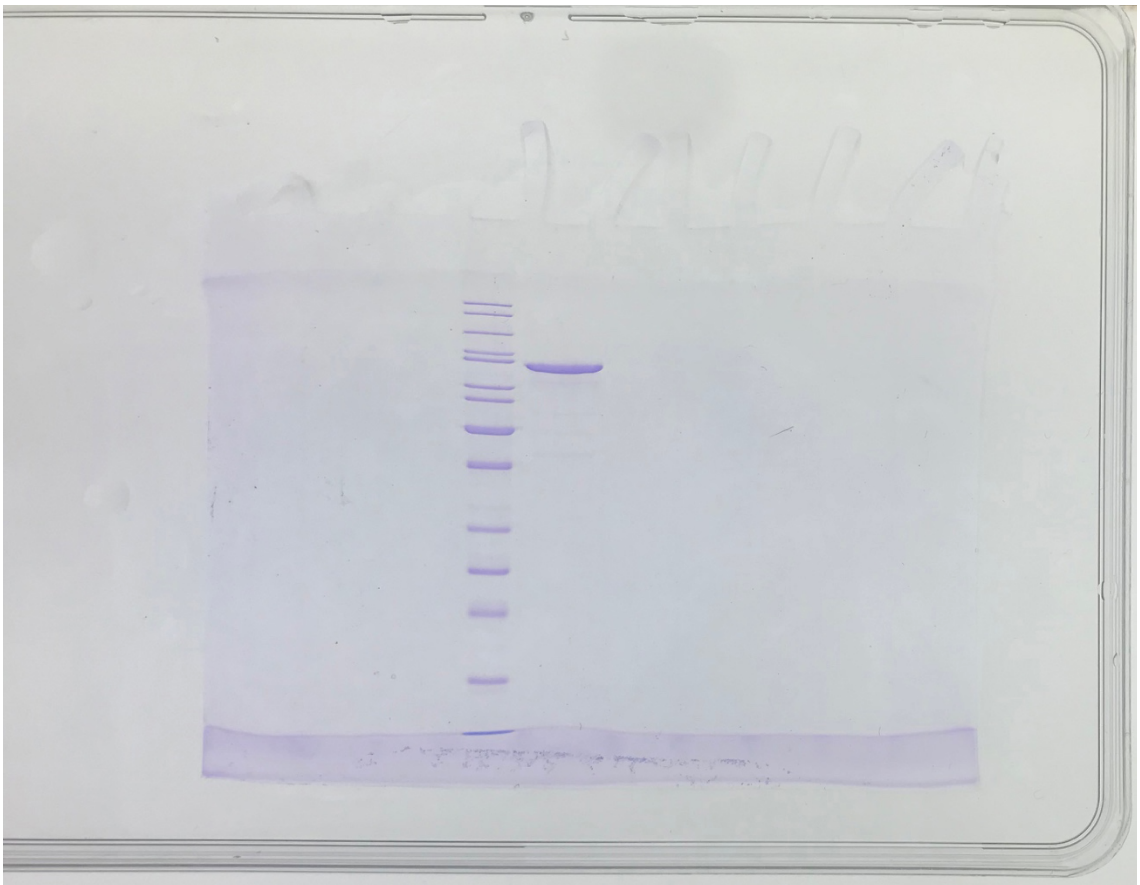

Supplementary Figure S1. Unprocessed image of SDS-polyacrylamide gel electrophoresis of purified PBOR_28850. The cropped gel image was used for Figure 1. The left and right lanes are standard of molecular mass and purified PBOR_28850, respectively.
